# AS MUCH AS I CAN PREVENT… I’M GOING TO DO IT: A QUALITATIVE ANALYSIS OF WAYS PEOPLE MANAGE DEMENTIA-RELATED ANXIETY

**DOI:** 10.1093/geroni/igae098.1724

**Published:** 2024-12-31

**Authors:** Molly Maxfield, Allie Peckham, Dara James, Rachel Koffer

**Affiliations:** Arizona State University, Phoenix, Arizona, United States; Arizona State University, Phoenix, Arizona, United States; Arizona State University, Tempe, Arizona, United States; Arizona State University, Phoenix, Arizona, United States

## Abstract

Alzheimer’s disease and related dementias (ADRD) are prevalent, without cures, and associated with declining independence. Beyond their cognitive effects, ADRDs can create social, psychological, and financial challenges for the individual diagnosed, their family, friends, and caregivers. Dementia-related anxiety (DRA) is the fear or anxiety about a current or future diagnosis of ADRD. The present study examined self-identified ways of managing DRA. Semi-structured qualitative interviews focused on thoughts and feelings about dementia among 50 community-dwelling adults (58 to 89 years old, M = 70.80, SD = 6.02) without dementia diagnoses. A reflexive inductive thematic approach examined ways people managed DRA. Based on an iterative process, we adopted and revised themes, ultimately identifying five themes: 1) monitoring cognitive status (e.g., self-monitoring or objective assessment), 2) active coping strategies (e.g., using external reminders, normalizing age-related change), 3) interpersonal relationships and support (e.g., anticipating benefit of support from others), 4) planning and preparing for potential outcomes (e.g., securing power of attorney and/or advance directives, saying goodbyes), and 5) personal responsibility to manage dementia risk or accept a diagnosis (e.g., lifestyle factors to reduce dementia risk, thereby reducing risk for burdening others). Findings suggest people use internal and external means for coping with DRA that are likely to vary in their usefulness. We consider these findings within the context of terror management theory and the lifespan theory of control, attending to potential clinical interventions for individuals experiencing DRA.

